# Benefits of Physical Exercise on Basic Visuo-Motor Functions Across Age

**DOI:** 10.3389/fnagi.2014.00048

**Published:** 2014-03-17

**Authors:** Marika Berchicci, Giuliana Lucci, Rinaldo Livio Perri, Donatella Spinelli, Francesco Di Russo

**Affiliations:** ^1^Department of Human Movement, Social and Health Sciences, University of Rome “Foro Italico”, Rome, Italy; ^2^Neuropsychological Unit, IRCCS Santa Lucia Foundation, Rome, Italy; ^3^Department of Psychology, University of Rome “La Sapienza”, Rome, Italy

**Keywords:** simple response task, response time, event-related potential, prefrontal cortex, lifespan

## Abstract

Motor performance deficits of older adults are due to dysfunction at multiple levels. Age-related differences have been documented on executive functions; motor control becomes more reliant on cognitive control mechanisms, including the engagement of the prefrontal cortex (PFC), possibly compensating for age-related sensorimotor declines. Since at functional level the PFC showed the largest age-related differences during discriminative response task, we wonder whether those effects are mainly due to the cognitive difficulty in stimulus discrimination or they could be also detected in a much easier task. In the present study, we measured the association of physical exercise with the PFC activation and response times (RTs) using a simple response task (SRT), in which the participants were asked to respond as quickly as possible by manual key-press to visual stimuli. Simultaneous behavioral (RTs) and electroencephalographic (EEG) recordings were performed on 84 healthy participants aged 19–86 years. The whole sample was divided into three cohorts (young, middle-aged, and older); each cohort was further divided into two equal sub-cohorts (exercise and not-exercise) based on a self-report questionnaire measuring physical exercise. The EEG signal was segmented in epochs starting 1100 prior to stimulus onset and lasting 2 s. Behavioral results showed age effects, indicating a slowing of RTs with increasing age. The EEG results showed a significant interaction between age and exercise on the activities recorded on the PFC. The results indicates that: (a) the brain of older adults needs the PFC engagement also to perform elementary task, such as the SRT, while this activity is not necessary in younger adults, (b) physical exercise could reduce this age-related reliance on extra cognitive control also during the performance of a SRT, and (c) the activity of the PFC is a sensitive index of the benefits of physical exercise on sensorimotor decline.

## Introduction

The proportion of adults over the age of 65 is expected to increase over the years. With advancing age, structural and functional deterioration occurs in most physiological systems, even in the absence of overt disease, including the central and peripheral nervous systems, as well as the neuromuscular system. A significant amount of new evidence has accumulated regarding the benefits of regular physical activity and exercise for older healthy adults (see Miller et al., 2012; Hayes et al., 2013, for reviews). Regular physical activity increases average life expectancy through its influence on sensorimotor control and functioning, and represents a low-cost, large-scale behavioral intervention that may slow the progression of physiological age-related cognitive and motor decline in healthy older adults.

In the last few years, neuroimaging studies have provided support for a positive correlation between cardiorespiratory fitness and cerebral structures and functions in humans (Colcombe et al., 2003; Gordon et al., 2008; Erickson et al., 2010; Bugg and Head, 2011; Weinstein et al., 2012), showing that brain structures, in particular the hippocampus and frontal and parietal areas mediate the positive association between fitness and cognition with particular emphasis on the executive functions of older adults. Intervention studies confirmed that aerobic exercise positively impacts the abovementioned brain structures (Colcombe et al., 2006; Erickson et al., 2011; Ruscheweyh et al., 2011; Voss et al., 2012). Other studies linked physical fitness to enhanced cognitive performance, which was mediated by the activity in most of the aforementioned areas in high-fit older adults (Colcombe et al., 2004; Godde and Voelcker-Rehage, 2010; Rosano et al., 2010; McGregor et al., 2011; Prakash et al., 2011; Smith et al., 2011; Voelcker-Rehage et al., 2011).

Despite the extensive neuroimaging literature on the effects of physical activity on brain health and cognitive functions, there is less electrophysiological evidence to date. It is well known that aging is associated with slowing of speed processing (Salthouse, 2000). The behavioral variable most frequently used to measure processing speed is the response time (RT) that is the interval between the onset of an external stimulus (e.g., visual, acoustic, etc.) and the manual response, such as key-press; when the RTs are recorded simultaneously to electroencephalographic (EEG) recordings, very rich information can be derived. The event-related potentials (ERPs) provide a description at high-temporal resolution of the various stages of motor preparation and information processing. ERPs represent a powerful tool to investigate the temporal dynamic of neural processing and, thus, provide clues to understand the neural basis of age-related slowing, and the cognitive strategy adopted by older adults. Differential slowing of separable ERP components can be linked to specific decline at sensory, motor, or cognitive level (see Daffner et al., 2013; Li et al., 2013; Stothart et al., 2013; Wiegand et al., 2013, for more details); this subtle discrimination is not possible using neuroimaging techniques. Some ERP studies focused on the brain activities preceding the stimulus onset during the motor preparation process. The results indicated that older adults were slower to react, because the pre-response processing were slowed and/or enhanced rather than perceptual processing (Yordanova et al., 2004; Roggeveen et al., 2007; Wild-Wall et al., 2007). However, fewer studies have investigated the relationship between physical fitness and electrophysiological activity in older adults. Hillman et al. (2004) recorded the ERPs during a visuo-motor discrimination task, and showed that physically active elderly have faster stimulus-related cognitive processing (as indicated by the latency of the parietal activity, measured by means of the well-known P3 component) and faster RTs than their sedentary peers. In our previous work, we reported that older adults engaged more prefrontal cortex (PFC) resources than middle-aged and young adults during response preparation in a visuo-motor discrimination task; this enable them to reach the same task accuracy as their younger counterparts, while their response speed was slower (Berchicci et al., 2012). We also showed that the participation at physical exercise programs could slow down the age-related cognitive decline from 35 to 40 years of age onward (Berchicci et al., 2013). In particular, we observed that physical activity was linked to enhanced executive performance during a go/no-go task mediated by decreased activity in prefrontal regions during movement preparation.

These findings indicate that increased age is associated with slower performance and enhanced recruitment of prefrontal areas in visuo-motor discrimination tasks; moreover, physical exercise could slow down this age-related cognitive decline. Since at functional level, the PFC, supporting executive control (Ridderinkhof et al., 2004), showed the largest age-related differences, we wonder whether the observed effects of physical activity were detectable only for difficult tasks, such as discriminative tasks, or they could also be detected in a much easier task not requiring discrimination. In the present study, we measured the relationship between physical exercise and both PFC activity and RTs using a simple response task (SRT). Based on previous findings, we expected to not observe exercise-related differences in cortical and behavioral measures of young adults; in contrast, the effects of physical exercise could be found in older adults. The investigation of middle-aged and older adults will tell us whether the PFC hyperactivity emerges also in a SRT; if this holds true, as reported by Berchicci et al. (2012), we could disconfirm the cognitive difficulty explanation reported in the literature (Yordanova et al., 2004) and consider the PFC hyperactivity as a generalized effect of aging. Further, if physical exercise could favor a decreased engagement of higher-order cortical structures also in a simple task, this could support the view that physical exercise is a non-pharmacological intervention able to reduce the age-related reliance on cognitive control. Finally, if the benefits of physical exercise in a SRT were confirmed at the PFC level but not at the level of pre-motor regions, then we can suggest that the PFC activity is a more sensitive electrophysiological index of motor decline with age.

## Materials and Methods

### Participants

A total of 84 participants volunteered. They were distributed across the following three adult age classes: younger (*n* = 30, 10 females, mean age = 24 years, age range: 19–35), middle-aged (*n* = 32, 12 females, mean age = 49 years, age range: 40–63), and older (*n* = 22, 14 females, mean age = 73 years, age range: 65–86). Education levels were similar in the two older groups (16.2 ± 2.1 and 16.0 ± 2.9 years of study for middle-aged and older, respectively) and slightly lower for younger adults (14.9 ± 1.6 years). Based on the self-report questionnaire about physical exercise (see below), the whole sample was equally divided into sub-cohorts: young adults who exercise (mean age: 24 ± 2.9 years) and do not exercise (mean age: 24 ± 5.4 years); middle-aged adults who exercise (mean age: 48 ± 3.8 years) and do not exercise (mean age: 51 ± 5.5 years); older adults who exercise (mean age: 74 ± 3.4 years) and do not exercise (mean age: 72 ± 4.2 years). To control potential confounds regarding gender and education level, the six sub-cohorts were preliminarily statistically compared. Results yielded not significant differences, indicating that they are matched for those factors. Furthermore, to control potential confounds regarding physical and mental health, only healthy people were included in the sample.

The general cognitive state of older participants was assessed using the mini-mental state examination (MMSE; Folstein et al., 1975). Older participants were not cognitively impaired, with an average MMSE score of 29 out of 30 (range: 28–30). All of the participants were healthy and without history of neurological, psychiatric, or chronic somatic problems. They were not taking psychoactive or vasoactive medication and had normal or corrected-to-normal vision. All of the participants were fully right-handed (Edinburgh Handedness Inventory; Oldfield, 1971). The older groups were recruited among friends of the authors and through the *Vitattiva Association* in Rome, the middle-aged participants were recruited among friends of the authors and among the employers of the University of Rome “Foro Italico,” and the younger participants were recruited from the local student population. The study received prior approval by the ethical committee of the IRCCS Santa Lucia Foundation. Written informed consent was obtained according to the Declaration of Helsinki from each participant.

### Physical activity assessment

Participants were asked to fill in a self-report questionnaire about physical exercise daily performed defined by the level of intensity in accordance with the American college of sport medicine guidelines (Chodzko-Zajko et al., 2009): moderate activity between three and six metabolic equivalent of tasks (METs) and vigorous activity greater than six METs. One MET is defined as the energy expenditure for sitting quietly, which, for the average adult, approximates 3.5 ml of oxygen uptake per kilogram of body weight per minute. Six METs was the threshold used to split the sample into not-exercise and exercise cohorts. The participants who reported general physical activities greater than six METs were also involved in regular physical exercise programs and sports at least 3 days/week, 1 h/session (i.e., swimming, running, martial arts, fencing, low- and high-impact exercise). Although the questionnaire approach may lead to omissions, inaccuracies, and bias, evidence has been reported about correlation between the results of subjective measures of physical activity and objective maximal oxygen consumption values (Bowles et al., 2004). Furthermore, the effects of physical fitness on cognitive and neural measures in older adults are consistent across different assessment methods (McAuley et al., 2011).

### Apparatus and procedure

The participants were tested after a 64-channel EEG active-cap was mounted on their scalp; they were seated in a darkened room in front of a screen placed 114 cm from their eyes. The visual stimuli were four squared configurations made by vertical and horizontal bars subtending 4° × 4° presented on a dark gray background; one of these stimuli was randomly displayed for 260 ms with equal probability (*p* = 0.25). The inter-stimulus interval varied randomly from 1 to 2 s in order to maintain uncertainty of the RT. The order of presentation was randomized within blocks. The duration of each run was 2.5 min with a pause interleaved; five runs allowed us to obtain 500 trials. A yellow circle (diameter 0.15° of visual angle) placed at the center of the screen and set at the participant’s eye level was the fixation point. Participants were asked to respond as fast as possible to all of the stimuli by pressing a button with the right index finger avoiding anticipations. Stimulus presentation and behavioral data acquisition were performed by Presentation™ software.

### Behavioral data analysis

Accuracy was measured by the percentage of anticipations (i.e., responses shorter than 100 ms or responses issued before the stimulus onset). The RTs’ medians for correct trials were calculated for each participant; the medians were used because the RTs’ means distributions are usually positively skewed, and the median is the appropriate measure under such conditions as long as RT differences are relevant (Baayen and Milin, 2010). The median RTs of each subject were then averaged using the mean for each sub-cohort. Factorial (3 × 2) ANOVAs were separately performed on the RTs and accuracy using the following factors: age (younger vs. middle-aged vs. older) and physical exercise (not-exercise vs. exercise). *Post hoc* comparisons were conducted using Tukey’s HSD test. The overall alpha level was fixed at 0.05 after the Geisser–Greenhouse correction.

### Electrophysiological recording and analysis

The continuous EEG was recorded using the BrainVision™ system with 64 active (ActiCap™) electrodes (BrainProducts GmbH, Munich, Germany) mounted according to the 10–10 International System, which were referenced to the left mastoid. The EEG was digitized at 250 Hz, amplified (bandpass of 0.01–80 Hz, including a 50 Hz notch filter), and stored for off-line averaging. Off-line analysis was performed utilizing the BrainVision™ analyzer 2.0.1 software (Brain Products GmbH, Munich, Germany). Raw EEG data were visually inspected to identify and discard epochs contaminated with artifacts prior to the signal averaging. The first trial of each block was discarded from further analysis. The trials with artifacts (e.g., blinks or gross movements) and amplitude exceeding threshold of ±120 μV were automatically excluded from the averaging, whereas eye movement artifacts were corrected using the Gratton et al. (1983) algorithm. Horizontal eye movements (electro-oculogram, EOG) were monitored with bipolar recordings from electrodes at the left and right outer canthi. The blinks and vertical eye movements were recorded with an electrode below the left eye, which was referenced to site Fp1. The participants were required to concentrate on the task performance and minimize distractions as much as possible. Possible sources of distraction and noise were minimized.

To comprehensively study the brain activity related to both response preparation and stimulus perception, EEG recordings were separately segmented and averaged into non-overlapping 2000-ms epochs that were measured from 1100 ms before to 900 ms after the stimulus onset. To further reduce high-frequency noise, the time EEG grand-averages were low-pass filtered at 25 Hz. The baseline was derived from the mean amplitude over the initial 200 ms of the averaged epochs. This approach allows investigating not only the post-stimulus ERP components [i.e., P1, prefrontal positivity (Pp), N1, P2, and P3], but also the pre-stimulus components related to the movement preparation [i.e., Bereitschaftspotential (BP) and prefrontal negativity (pN)], since the baseline was calculated from more than 1 s before the stimulus onset and not 100–200 ms right before the stimulus onset, as usually done. The mean amplitude in the −500/0 ms time window, reflecting activity during the pre-stimulus preparation stage, has been selected for further analysis on the following electrodes: Cz (roughly overlaying pre-motor and motor areas) for the BP component; Fp1 and Fp21 (over the PFC) for the pN component. After stimulus onset, peak amplitudes and latencies of the major ERP components were calculated for each subject in the following standard time windows: P1: 80–150 ms; pP: 80–120 ms; N1: 130–200 ms; P2: 180–300 ms; and P3: 250–700 ms. The electrodes selection was based on the scalp topography which allowed identifying the greatest activity for a given component at the group level (i.e., the P1 and P2 on PO7 or PO8, the pP on Fp1 or Fp2, the N1 on O1 or O2, the P3 on Pz) and on previous reports (e.g., Shibasaki and Hallett, 2006; Berchicci et al., 2012).

Factorial (3 × 2) ANOVAs were separately performed on the mean amplitude in the −500/0 ms time windows (pN and BP components) and on the peak latency and amplitude of the aforementioned post-stimulus components. Factors were: age (younger vs. middle-aged vs. older) and physical exercise (not-exercise vs. exercise). *Post hoc* comparisons were conducted using Tukey’s HSD test. The overall alpha level was fixed at 0.05 after the Geisser–Greenhouse correction. The correlation coefficients (Pearson’s *r* coefficients) were separately computed for exercise and not-exercise cohorts between the RTs and mean activity preceding the stimulus onset over central (BP component) and prefrontal (pN component) derivations. The significance was set at 0.05 (two-tailed) for all of the analyses.

To visualize the voltage topography of the ERP components, spline interpolated three-dimensional maps were constructed using the BESA 2000 software (MEGIS Software GmbH, Gräfelfing, Germany).

## Results

### Behavioral results

The accuracy and mean response times for the sub-cohorts are presented in Table 1. Older adults responded significantly slower than middle-aged and younger adults did, while not committing a greater number of anticipations (anticipation rates were below 1% and not differed between cohorts; all *p*s > 0.05). The factorial ANOVA did not reveal a significant age × physical exercise effect interaction (*F*_2,78_ = 0.03, *p* = 0.968, η^2^ = 0.000). There was a main effect of age on the RTs (*F*_2,78_ = 17.18, *p* < 0.0001, η^2^ = 0.305). Younger participants were on average 22 ms faster than middle-aged (*p* = 0.004) and 47 ms faster (*p* = 0.0001) than older participants, who were on average 25 ms slower (*p* = 0.002) than middle-aged. The RTs during this SRT were not significantly affected by physical exercise habits (*F*_2,78_ = 2.54, *p* = 0.114, η^2^ = 0.031); however, Table 1 indicates that, on average, exercise cohorts were 10 ms faster than not-exercise cohorts.

**Table 1:** **Mean and standard deviation (Mean ± SD) of the response times (RT) and anticipations (An) for each sub-cohort**.

Cohorts	Not-exercise		Exercise	
	RT (ms)	An (%)	RT (ms)	An (%)
Young	211 ± 19	0.31 ± 0.07	200 ± 20	0.21 ± 0.06
Middle-aged	233 ± 38	0.15 ± 0.04	221 ± 32	0.18 ± 0.05
Older	256 ± 31	0.30 ± 0.04	248 ± 30	0.22 ± 0.09

### Electrophysiological results

**Figure 1:**
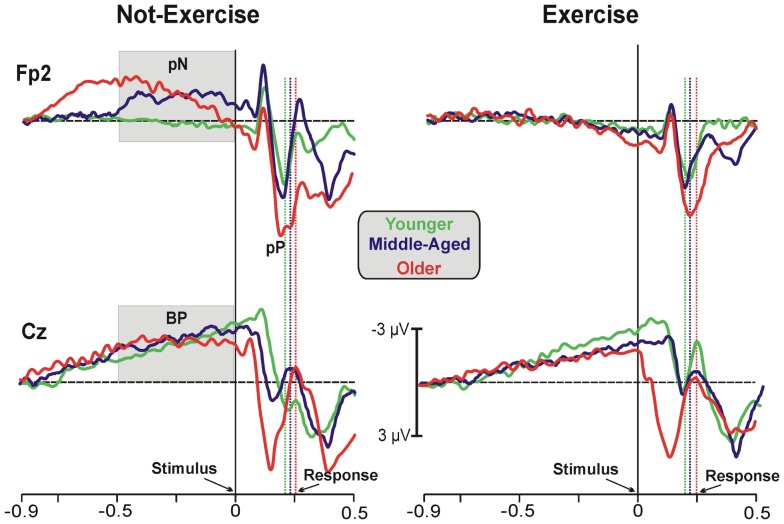
**Grand-average ERP waveforms at the prefrontal (Fp2) and central (Cz) electrodes**. The age groups are superimposed with different colors (see the legend in the figure) and divided between not-exercise (left) and exercise (right) cohorts. The time zero represents the stimulus appearance; the vertical dotted lines represent the response emissions for each cohort, based on the color of the ERP waveforms.

Figure 1 shows the grand-averaged ERP waveforms at the representative prefrontal (Fp2) and central (Cz) sites, where the pN and BP components were maximal. Waveforms of the three age groups were superimposed and separately displayed for not-exercise and exercise cohorts. The activity over prefrontal sites was modulated by both age and exercise. Vertical dotted lines represent the RTs in each cohort. Within the not-exercise cohorts, the waveform of the young adults was slightly positive before the stimulus onset, whereas those of the middle-aged and older adults showed a slow rising negativity (pN), which began very early in the older cohort. Within the exercise cohorts, the pN of the three age groups was absent (did not differ from baseline). After stimulus onset, another main component over the PFC was detectable: the pP. The peak latency was approximately 196 ms in all of the cohorts independently from physical exercise, whereas pP amplitude increased with age. The BP component was very similar in all of the cohorts over the central derivation (Cz). This component showed the typical slow rising negativity that reflects motor preparation. This negativity reached the peak at approximately 100 ms after the stimulus onset.

Statistical analysis of the pN amplitude showed a main effect of physical exercise (*F*_1,78_ = 6.17, *p* = 0.015, η^2^ = 0.073). The ANOVA did reveal a significant age × physical exercise effect interaction (*F*_2,78_ = 3.12, *p* = 0.045, η^2^ = 0.074). *Post hoc* analysis showed that the participants who exercise did not recruit the PFC to accomplish this SRT regardless of the age group, whereas the older (−2.23 ± 0.9 μV) and middle-aged adults (−1.86 ± 0.6 μV) with a more sedentary lifestyle needed the intervention of the PFC control also in this very easy task. ANOVA on the pP amplitude did not reveal a significant age × physical exercise effect interaction, but there was a main effect of age (*F*_2,78_ = 6.59, *p* = 0.002, η^2^ = 0.172), with larger pP amplitude in older (6.43 ± 0.5 μV) than middle-aged (4.32 ± 0.4 μV) and younger (4.04 ± 0.4 μV) adults. Physical exercise did not significantly affect the pP amplitude (*F*_1,78_ = 1.38, *p* > 0.05, η^2^ = 0.016), even thought the exercise cohorts showed lower amplitude (4.81 ± 0.3 μV) than not-exercise (5.48 ± 0.3 μV) cohorts. The pP latency was not different across cohorts. Statistical analysis of the BP amplitude did not yield significant results. Similarly, analysis on the P1, N2, and P2 peak latency and amplitude were not significant. ANOVA on the P3 latency showed a main effect of age (*F*_2,78_ = 8.059, *p* = 0.006, η^2^ = 0.120), with later P3 in older (417 ± 75.80 ms) than middle-aged (363 ± 77.68 ms) and younger (356 ± 87.65 ms) adults. The physical exercise effect and the interaction were not significant. Not significant effects were found on the P3 amplitude.

**Figure 2:**
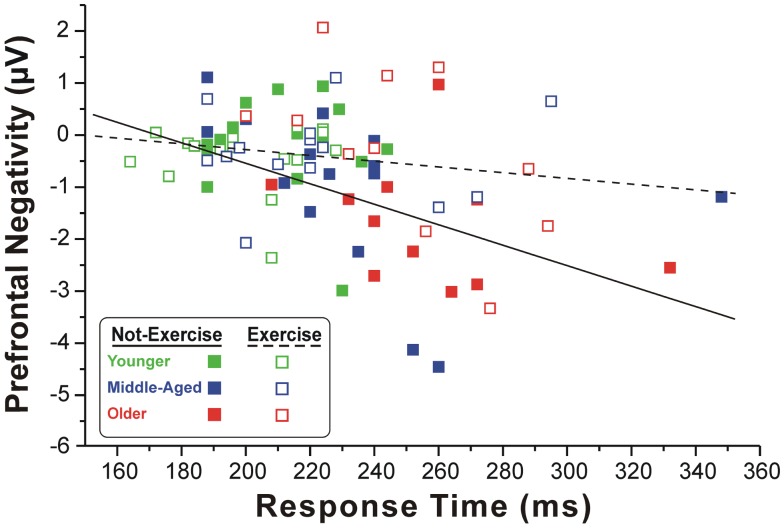
**Scatter plot of the correlation between the RTs and pN component in the two cohorts of exercise and not-exercise participants**. The dashed line represents the linear fit of the exercise cohort, whereas the solid line refers to the not-exercise cohort. Only the former is significant, showing that larger is the PFC activity and slower is the RT.

Figure 2 shows the scatter plot of the correlations between the RTs and the pN component on Fp2, which was the most representative site, for both exercise and not-exercise cohorts. A significant negative correlation was found in the not-exercise cohort (continuous line in the figure; *R* = −0.48; *p* = 0.001), whereas no correlation was observed in the participants that exercise (dashed line in the figure; *R* = −0.17; *p* = 0.277). Removing the two outliers (filled squares RTs: 332 and 348 ms) from middle-aged and older adults that do not exercise, the correlation was even stronger (*R* = −0.55; *p* = 0.001). No correlation was found between RTs and the BP component parameters.

### Topographical maps

**Figure 3:**
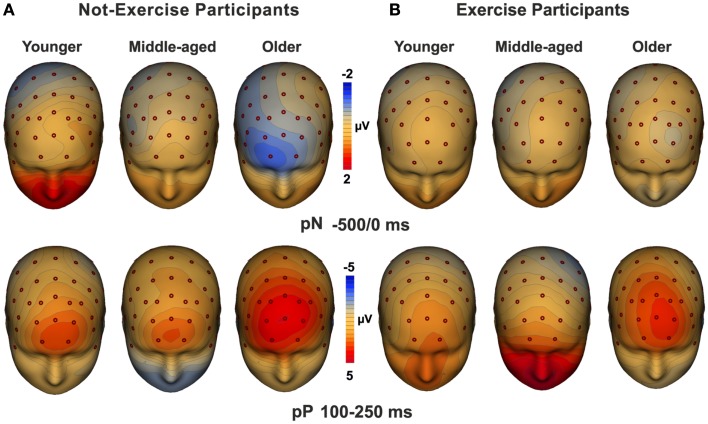
**Topographical maps of the pN (up) and pP (bottom) components**. The maps for young, middle-aged, and older adults are displayed from the left to the right for not-exercise **(A)** and exercise **(B)** cohorts.

Three-dimensional topographical maps of the grand-averaged data for the pN and the pP components are displayed in Figure 3. The maps of younger, middle-aged, and older participants are displayed from the left to the right for not-exercise cohorts (a) and exercise cohorts (b). The top row maps show the negative activity over the PFC in the −500/0 ms time window, which was present only in the older not-exercise adults. The bottom row maps show the topography of the pP when it was maximal. The positivity was very large in older adults, but it was also visible in middle-aged and younger adults that do not exercise. The maps of the physical exercise cohorts show the smaller pP than not-exercise in all of the age groups.

## Discussion

This study examined the effects of active lifestyle on PFC activity and RTs across ages using a simple response task. The results indicate that: (a) the PFC control is needed also for a very simple task in older adults, while it is not necessary in younger adults, (b) physical exercise could reduce this age-related reliance on additional cognitive control, (c) age-related decline and physical exercise did not affect the activity of pre-motor and visual areas, and (d) as known, parietal areas were affected by age (delay of the P3 component), but not by physical exercise.

Task difficulty is a confounding factor in studies investigating sensorimotor changes with advancing age. Indeed, discrimination increases task difficulty, as indicated by errors increment and, remarkably, much longer RTs than in the SRT in all of the age groups (Berchicci et al., 2012). There is abundant evidence for a decreased efficiency of motor neuron recruitment in elderly that can be compensated by increased involvement of prefrontal cortical resources as a function of task difficulty, because the same task tend to be more demanding for older than younger adults (Stewart et al., 2013). Thus, older adults use compensatory mechanisms in order to maintain a performance level comparable to their younger counterparts (Reuter-Lorenz and Cappell, 2008) except for the slowing of RTs. However, present and past studies (Berchicci et al., 2012) showed a prefrontal hyperactivity also when the discrimination factor was excluded.

A study combining ERP and fMRI measures localized this preparatory prefrontal activity in the inferior frontal gyrus (iFg) of both hemispheres (Di Russo et al., 2013). Other fMRI studies using a SRT confirmed enhanced activity of prefrontal regions in elderly compared with younger participants (Sailer et al., 2000; Davis et al., 2008). Transcranial magnetic stimulation (TMS) studies showed that the excitability of corticospinal pathways of older adults is tuned in advance to speed up response generation and enhance the response threshold of the ipsilateral cortex (Levin et al., 2011); this preparatory strategy could increase the readiness for fast and accurate response (Sinclair and Hammond, 2009). In the present study, although the task difficulty was minimal, older adults were slower than younger and middle-aged adults. Note also the remarkable difference in the RTs between young and middle-aged adults, confirming that the slowing of processing is detectable already in middle-aged, compromising activities of daily living (such as driving, crossing the street, grasping an object that is falling down, etc.), social interactions, decision making and learning (Birren and Fisher, 1995). Further, the RTs of the participants that have a more physically active life style tend to be shorter than their less active counterparts (see Table 1), although the difference did not reach significant level. Interestingly, in not-exercise cohort, we found a negative correlation between the RTs and pN amplitude, likely mediated by the iFg and reflecting the cognitive preparation of the response. The recruitment of the iFg increases with age, as well as the RTs: the larger the activity in prefrontal structures before the stimulus onset, the slower was the RT. On the other hand, the physical exercise reduced the age-related recruitment of the iFg and this was associated with faster RTs.

Another prefrontal activity characterized by positive polarity (the pP component) started about 100 ms after stimulus onset and reached the peak concomitantly to the key-press in young adults and few milliseconds before the response in older adults. The pP was localized in the anterior Insula and was considered as a process of sensory evidence accumulation up to reaching a threshold for motor response (Di Russo et al., 2013). The pP was larger in older than younger adults, but it was not modified in a significant way by physical exercise, although the topographical maps suggest this trend. Considering this activity as a process of sensory evidence accumulation for response execution, older people, and in part also middle-aged adults, need more time than younger adults to reach the response threshold, with a concomitant slowing in processing speed: this could make the person (i.e., elderly and sedentary) behaviorally slow, according to the literature on age-related slowing of nerve conduction (Macaluso and De Vito, 2004), and would explain the greater engagement of the brain structures involved. This conclusion is in line with other studies, showing a greater insular activation with increased task difficulty (Philiastides and Sajda, 2007) and aging (Williamson et al., 1999).

The activity in pre-motor and visual regions during this SRT was affected neither by age nor by physical exercise. As previously found (e.g., Berchicci et al., 2012), the age-related delay in response emission and categorization (indexed by the parietal P3) was confirmed, but no effects of physical exercise were found. This latter result is only partially in line with previous studies, because Hillman et al. (2004) showed that the P3 latency was modulated by physical exercise. However, they found differences in the P3 latency only between high physically active older adults and the other groups (moderate and low active participants); in the present study there are only two groups, and this methodological difference could explain the lack of differences between exercise and not-exercise cohorts. It is worth noting that one limitation of the present study is the size of the sample, which is relatively small especially for the older cohorts. Indeed, lack of significance on the P3 latency and at behavioural level could also be explained by the small size of the sub-cohorts analysed.

Meta-analyses of physical activity interventions confirmed that the effect of exercise on cognitive functions is both general and specific (Erickson et al., 2013). It is general because different cognitive domains are positively affected by exercise, regardless of the physical activity and fitness assessment used (Liu-Ambrose et al., 2012). It is specific because the executive functions are more improved by exercise interventions than other functions (Colcombe and Kramer, 2003; Hillman et al., 2008). This suggests that brain regions and networks supporting executive functions might be more sensitive to the effects of exercise than other brain areas. Changes in the frontal cortical areas cause the decline of cognitive processes associated with executive and attentional functioning, while other cognitive processes which do not rely on these brain areas, such as implicit memory, verbal ability, and word knowledge, remain relatively stable across lifespan (Ballesteros et al., 2013); these latter functions are also less affected by physical exercise. One possible explanation is that the PFC is more plastic than other brain regions (Van Praag et al., 2005; Kempermann et al., 2010). The ability to mitigate age-related motor and cognitive decline is critical for a successful aging (Seidler et al., 2010) and the physical exercise interventions hold great promise in this regard (Miller et al., 2012). Present findings expand previous studies, demonstrating that the beneficial effects of physical exercise are detectable at brain level also during an extremely simple response task, but were not indeed evident at behavioral level. This kind of task could be a handy tool suitable to investigate the effectiveness of training programs and to verify the outcomes of rehabilitation interventions, because it allows to assess both behavioral and cortical age-related changes with physical exercise. In particular, the behavioral variables are sensitive to sensorimotor age-related decline, but are not completely responsive to the exercise-related changes, which could be well disclosed using ERPs analysis. Furthermore, future studies using self-paced paradigms would also improve our knowledge about the exercise-induced modulation on spontaneous actions which are not activated by external events.

## Conflict of Interest Statement

The authors declare that the research was conducted in the absence of any commercial or financial relationships that could be construed as a potential conflict of interest.
